# An antibody-free LC-MS/MS method for the quantification of intact insulin-like growth factors 1 and 2 in human plasma

**DOI:** 10.1007/s00216-021-03185-y

**Published:** 2021-02-10

**Authors:** Mark S. Pratt, Martijn van Faassen, Noah Remmelts, Rainer Bischoff, Ido P. Kema

**Affiliations:** 1 Department of Laboratory Medicine, University Medical Center Groningen, University of Groningen, Hanzeplein 1, 9713 GZ Groningen, The Netherlands; 2 Department of Analytical Biochemistry, Groningen Research Institute of Pharmacy, University of Groningen, Antonius Deusinglaan 1, 9713 AV Groningen, The Netherlands

**Keywords:** Insulin-like growth factor 1 (IGF-1), Insulin-like growth factor 2 (IGF-2), Liquid chromatography-tandem mass spectrometry (LC-MS/MS), Peptide hormone analysis, Biomarker

## Abstract

Insulin-like growth factors 1 and 2 (IGF-1 and IGF-2) are important biomarkers in research and diagnosis of growth disorders. Quantitative analysis is performed using various ligand-binding assays or enzymatic digestion LC-MS/MS methods, whose widespread adoption is hampered by time-consuming sample preparation procedures. We present a simple and fast antibody-free LC-MS/MS method for the quantification of intact IGF-1 and IGF-2 in human plasma. The method requires 50 μL of plasma and uses fully ^15^N-labelled IGF-1 as internal standard. It features trifluoroethanol (TFE)-based IGF/IGF-binding protein complex dissociation and a two-step selective protein precipitation workflow, using 5% acetic acid in 80/20 acetone/acetonitrile (precipitation 1) and ice-cold ethanol (precipitation 2). Detection of intact IGF-1 and IGF-2 is performed by means of a Waters XEVO TQ-S triple quadrupole mass spectrometer in positive electrospray ionisation (ESI+) mode. Lower limits of quantification were 5.9 ng/mL for IGF-1 and 8.4 ng/mL for IGF-2. Intra-assay imprecision was below 4.5% and inter-assay imprecision was below 5.8% for both analytes. An excellent correlation was found between nominal and measured concentrations of the WHO reference standard for IGF-1. Comparison with the IDS-iSYS IGF-1 immunoassay showed good correlation (*R*^2^ > 0.97), although a significant bias was observed with the immunoassay giving substantially higher concentrations. The LC-MS/MS method described here allows for reliable and simultaneous quantification of IGF-1 and IGF-2 in plasma, without the need for enzymatic digestion. The method can be readily implemented in clinical mass spectrometry laboratories and has the potential to be adapted for the analysis of different similarly sized peptide hormones.

**Supplementary Information:**

The online version contains supplementary material available at 10.1007/s00216-021-03185-y.

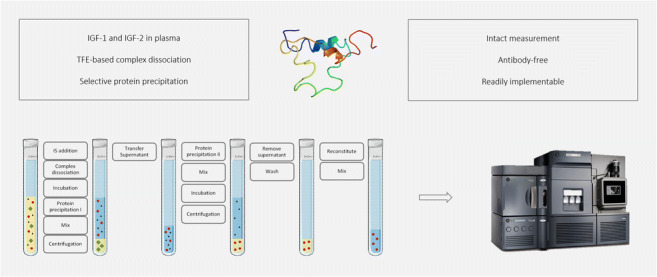

## Introduction

Insulin-like growth factor 1 (IGF-1, MW 7649 Da) and insulin-like growth factor 2 (IGF-2, MW 7470 Da) are polypeptide hormones, similar in molecular structure to insulin. Physiologically they act as the main mediators of growth hormone (GH)-stimulated cell and tissue growth. Despite the fact that they do not originate from the same genetic locus, both IGFs exert their main physiological effects by binding to the IGF-1 receptor. Though in contrast to IGF-1, which plays an important role in childhood growth and continues to have anabolic effects in adults, the major physiological role of IGF-2 is as a growth-promoting hormone during gestation [1, 2]. In laboratory medicine, IGF-1 has various applications [2–7], including the diagnosis and monitoring of growth hormone–related disorders (e.g. dwarfism, gigantism or acromegaly), whereas in some cases, IGF-2 is produced in excess in islet cell tumours and non-islet hypoglycaemic cell tumours, making it an interesting biomarker in these situations [8, 9].

Quantitative analysis of both IGF-1 and IGF-2 is traditionally performed using different ligand-binding assays (LBAs). However, these IGF immunoassays tend to suffer from intra- and interlaboratory variation and poor reproducibility due to their dependence on antibodies, for which significant batch-to-batch variability has been observed [10–14]. In recent years, liquid chromatography coupled to tandem mass spectrometry (LC-MS/MS) has emerged as an alternative platform for the quantification of proteins in complex biological matrices [15–24]. Given the unique ability of mass spectrometry to use stable-isotope-labelled internal standards combined with highly specific detection based on mass-to-charge ratios, this technique has the possibility to separate and detect different forms of the same protein, is less prone to interferences and is characterised by an increased (interlaboratory) reproducibility compared to LBAs.

In order to reach sufficient analytical sensitivity for the quantitative analysis of proteins, the majority of LC-MS/MS methods for IGF-1 rely on immunoaffinity techniques as a mode of sample clean-up [15–17] and are performed at the peptide level [17–23]. This workflow, where, following enzymatic digestion, a unique peptide serves as surrogate for the protein is aimed at combating sensitivity-related issues that arise in protein mass spectrometry, such as poor transmission and fragmentation of intact protein ions. A disadvantage of this workflow, however, is that these techniques are generally relatively time-consuming, making it difficult to implement these methods into clinical laboratories. Furthermore, despite the use of stable-isotope-labelled internal standards, small variations in enzymatic digestion conditions may cause significant differences in analytical results [25]. Alternatively, quantitative IGF-1 assays based on high-resolution mass spectrometry have been described [16, 26–29]. However, this equipment may not be readily available in clinical laboratories.

In this work, we describe an LC-MS/MS method for the simultaneous quantification of intact endogenous IGF-1 and IGF-2 in human plasma that can be readily implemented in clinical mass spectrometry laboratories. The method introduces a two-step selective protein precipitation strategy that can be largely automated, and does not rely on immunoaffinity sample clean-up or enzymatic digestion steps.

## Materials and methods

### Materials

Recombinant human IGF-1 (cat. no. cyt-216), IGF-2 (cat. no. cyt-265), ^15^N-IGF-1 (cat. no. cyt-128) and IGF-binding protein 3 (IGFBP-3, cat. no. cyt-300) were obtained from ProSpec (Ness-Ziona, Israel) and were all produced in *E. coli*. The WHO international standard 02/254 for IGF-1 [30] was acquired from the National Institute for Biological Standards and Control (NIBSC, South Mimms, UK). Rat plasma was purchased from BioIVT (Burgess Hill, UK). UPLC-MS grade acetonitrile (ACN) and formic acid (FA), as well as LC-MS grade methanol (MeOH) and isopropanol (IPA) and AR grade acetone and ethanol, were obtained from Biosolve (Valkenswaard, The Netherlands). Ovalbumin, 2,2,2-trifluoroethanol (TFE) and acetic acid (HAc) were acquired from Sigma-Aldrich (Zwijndrecht, The Netherlands).

#### Calibrants

A vial containing 1000 μg of IGF-1 was reconstituted in 1000 μL 2% (w/v) ovalbumin in phosphate-buffered saline (PBS) and subsequently diluted in 2% ovalbumin in PBS to a concentration of 285 μg/mL. Ovalbumin was used to prevent non-specific adhesion of IGFs to the container and any potential contamination of ovalbumin with chicken IGF-1 or IGF-2 will not yield a signal due to sequence variation. For IGF-2, 50 μg was dissolved in 250 μL 2% ovalbumin in PBS to obtain a 200 μg/mL solution. These IGF-1 and IGF-2 solutions were then diluted and combined to form two stock solutions containing (1) 95 μg/mL of IGF-1 and 133 μg/mL of IGF-2 and (2) 9.5 μg/mL of IGF-1 and 13.3 μg/mL IGF-2. By diluting these stock solutions in rat plasma, calibrants were obtained at 14, 28, 56, 190, 379, 756 and 1129 ng/mL IGF-1 and 20, 40, 80, 266, 531, 1058 and 1581 ng/mL IGF-2.

Fully ^15^N-labelled IGF-1 internal standard was reconstituted in 2% ovalbumin in PBS to obtain a 10 μg/mL stock solution. This stock was then further diluted in 50% trifluoroethanol in water to a final concentration of 200 ng/mL.

Pooled anonymised human EDTA plasma collected from routine patient care was used for quality control (QC) samples either directly to obtain QC low, or spiked with IGF standards to obtain QC medium (+ 200 ng/mL IGF-1 and + 550 ng/mL IGF-2) and QC high (+ 575 ng/mL IGF-1 and + 925 ng/mL IGF-2), thus resembling expected plasma concentrations. This was classified as non-WMO research (Dutch Medical Research Involving Human Subjects Act), and received an exemption from the Medical Ethical Committee of our hospital. Internal standard solution and quality control samples were stored at − 20 °C, whereas calibrants were stored at − 80 °C until analysis.

#### Sample preparation

Blood samples were collected by venipuncture using EDTA Becton Dickinson (Franklin Lakes, USA) Vacutainer Tubes. After centrifugation (4 °C, 2500*g*, 10 min), plasma was transferred into plastic tubes within 4 h of collection and stored at − 20 °C until analysis.

**Fig. 1:**
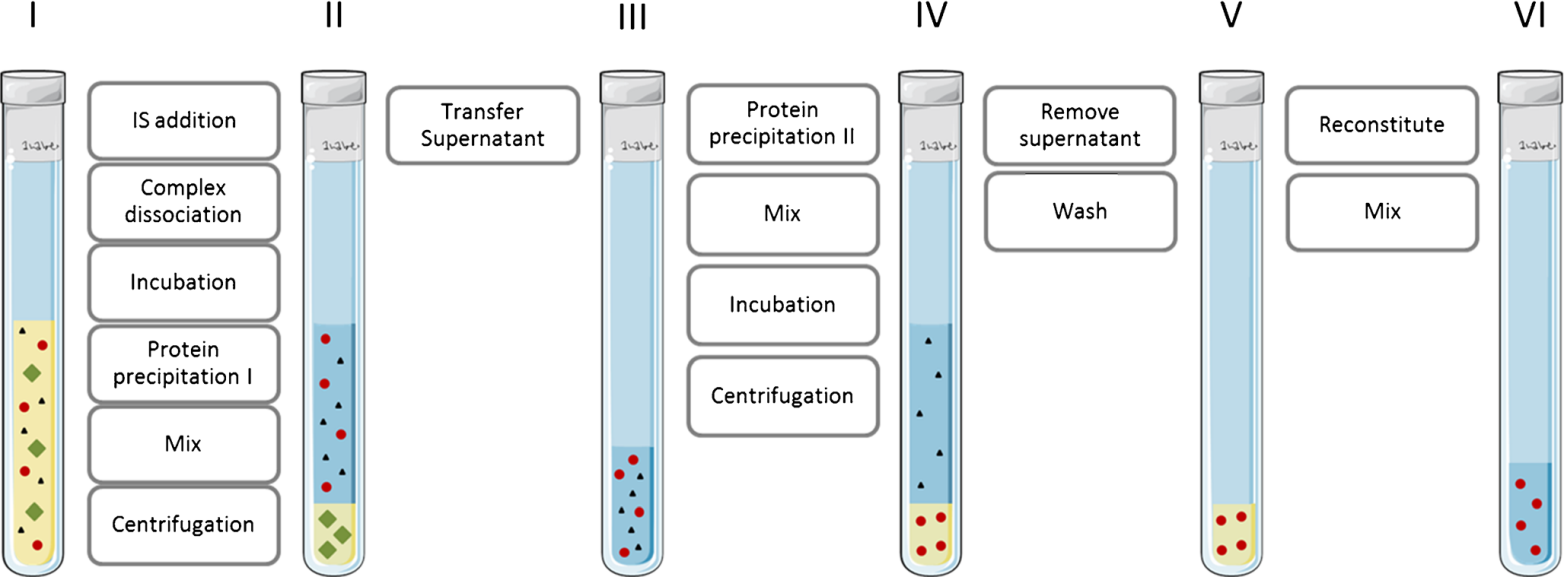
Selective precipitation workflow. Red: peptide hormones (including IGF-1 and IGF-2); green: large proteins (> 20 kDa); black: lipids and salts. To raw plasma (I) internal standard, carrier protein complex dissociation reagent and the first precipitation reagent are added, resulting in the precipitation of large high-abundant proteins, whereas IGF-1 and IGF-2 remain in solution (II). The supernatant is then transferred to a new well (III) and subsequently the second precipitation reagent is added and the sample is incubated at − 20 °C for 45 min, resulting in the precipitation of small proteins (IV). Interfering salts and lipids are removed from the sample by discarding the supernatant and washing the pellet, leaving only the precipitated proteins (V). This pellet is then reconstituted ready for injection onto the LC-MS/MS (VI)

For analysis, 50 μL of plasma was transferred into a 700-μL round-well plate (Waters, Milford, USA). IGF/IGFBP complexes were dissociated and proteins were denatured by the addition of 75 μL of 50% (v/v) TFE in water containing 200 ng/mL ^15^N-IGF-1, followed by 15 min incubation [31]. Subsequently, 100 μL of 5% (v/v) acetic acid in 20% (v/v) acetonitrile in acetone was added in order to precipitate high-abundance large proteins (selective precipitation, optimised to keep IGFs in solution) [32]. The samples were vortex-mixed and centrifuged for 10 min (4 °C, 2500*g*), following which 125 μL of the supernatant was transferred to a deep-well plate (Greiner Bio-One, Kremsmünster, Austria). A second precipitation was performed by adding 1000 μL of ethanol (− 20 °C), with the aim of precipitating smaller proteins (including IGF-1 and IGF-2) [33]. The samples were then vortex-mixed, incubated for 45 min at − 20 °C and centrifuged for 30 min (4 °C, 2500*g*). The supernatant was removed and the pellet was washed with 1000 μL ethanol (− 20 °C). Finally, the pellet was reconstituted in 90 μL 10% (v/v) acetonitrile containing 1% (v/v) formic acid in water before injection onto the LC-MS/MS system. A schematic representation of the full workflow from raw plasma to injection is provided in Fig. 1. Total sample preparation time was below 3 h for a single batch of plasma samples.

#### LC-MS/MS analysis

Samples were analysed using a Waters Acquity I-Class liquid chromatography system with a flow-through needle (FTN) autosampler. 5.0 μL was injected onto a Phenomenex (Torrance, USA) Luna Omega Polar C18 column (1.6 μm particle size, 2.1 × 100 mm) kept at 60 °C. Gradient elution was performed at 0.4 mL/min using 0.1% (v/v) formic acid in water (mobile phase A) or in acetonitrile (mobile phase B) as follows: 0–5.5 min linear gradient from 5 to 30% B; 5.5–6.0 min linear gradient from 30 to 40% B; 6.0–6.1 min linear gradient from 40 to 50% B; column flush from 6.1–7.0 min at 50% B; column re-equilibration from 7.0–8.5 min at 5% mobile phase B.

Detection was performed by means of a Waters XEVO TQ-S triple quadrupole mass spectrometer in multiple reaction monitoring (MRM) mode, using positive electrospray ionisation (ESI+). Capillary voltage was maintained at 2500 V, cone voltage at 30 V and desolvation temperature at 650 °C. Nitrogen was used as desolvation gas (1100 L/h) and cone gas (150 L/h), whereas argon was used as collision gas. An overview of MRM transitions is presented in Table 1.

**Table 1:** MS/MS parameters. Product ion scans, along with the amino acid sequences of both the precursor and product ions are shown in Supplemental Figs. S1 to S5

	Precursor ion (*m/z*)	Collision energy (eV)	Product ion (*m/z*)
Analytes			
IGF-1 (quantifier)	956.95 [M + 8H]^8+^	29	1175.55 (b_64_^6+^)
IGF-1 (qualifier)	1093.45 [M + 7H]^7+^	33	1196.95 (b_65_^6+^)
IGF-2 (quantifier)	934.50 [M + 8H]^8+^	27	1057.90 (y_66_^7+^)
IGF-2 (qualifier)	934.50 [M + 8H]^8+^	28	989.70 (b_62_^7+^ − H_2_O)
Internal standard			
^15^N-IGF-1 (quantifier)	968.50 [M + 8H]^8+^	29	1189.70 (b_64_^6+^)
^15^N-IGF-1 (qualifier)	1106.65 [M + 7H]^7+^	33	1211.45 (b_65_^6+^)

System operation and data acquisition were controlled using Waters MassLynx 4.0 and data was processed using TargetLynx 4.1 software.

#### Assay characteristics

In order to assess the effectiveness of the trifluoroethanol-based carrier protein dissociation, an IGF-binding protein challenge test was carried out by spiking 45 plasma samples with a 10-fold molar excess of IGFBP-3, followed by 45 min incubation. The samples were then analysed according to the LC-MS/MS method detailed here and results were compared to the results of the non-spiked samples. This methodology is in line with previous publications [19, 22, 27].

Bicinchoninic acid assay (BCA) analysis was carried out in order to determine the total protein concentration following sample preparation, and thus to estimate the efficacy of the selective precipitation clean-up workflow.

#### Method validation and comparison

Analytical validation for both IGF-1 and IGF-2 was performed based on the guidelines for bioanalytical method validation from the Dutch Coordinating Commission for Quality Management in Medical Laboratories (CCKL) and the ISO 15189:2012 standard [34].

Ionisation suppression at the retention times of IGF-1 and IGF-2 was assessed by means of a post-column infusion experiment. Linearity of the method was determined by analysing calibration curves on six different days. Curves were plotted using least-squares linear regression and checked for linearity. In order to determine whether a matrix effect was present, a low concentration QC sample was mixed with a high concentration QC sample in different ratios. These sample mixtures were then analysed and their linearity was assessed.

Carry over was estimated through the analysis of alternating injections of low concentration samples (55 ng/mL IGF-1, 458 ng/mL IGF-2) and high concentration samples (667 ng/mL IGF-1, 1469 ng/mL IGF-2).

The lower limit of quantification (LLOQ) was established from six replicates of serially diluted plasma samples. The LLOQ was defined as the concentration at which the coefficient of variation was equal to 20%. Intra-assay variation was determined by analysing 10 replicates of pooled samples of low, medium and high concentrations of IGF-1 and IGF-2 on the same day. For the determination of the inter-assay variation, three pooled samples were analysed on 10 different days. Recovery was assessed by spiking two fresh plasma samples at three different concentration levels. These samples were then analysed on six different days, and the recovery was determined as follows: [(final concentration − initial concentration)/added concentration] × 100%. Autosampler stability at 10 °C was assessed at 24 h, 48 h, 72 h and 7 days.

Accuracy of the method was assessed by reconstituting the WHO international standard for IGF-1 in 2% (w/v) ovalbumin in PBS in two-fold and subsequent dilution of both vials in rat plasma to nominal concentrations of 850 ng/mL and 85 ng/mL in five-fold. These ten aliquots were then processed over five different runs according to the validated workflow described above and measured concentrations were compared to the nominal concentrations. Furthermore, the method was compared to the IDS-iSYS IGF-1 immunoassay using 72 anonymised clinical samples across the clinically relevant concentration range, which were obtained from left-over routine patient care samples (also classified as non-WMO research, thus receiving an exemption from the Medical Ethical Committee). As the immunoassay is not compatible with plasma samples, the comparison was carried out using serum samples. No differences from plasma to serum were observed for the LC-MS/MS detailed here (data not shown).

## Results

**Fig. 2:**
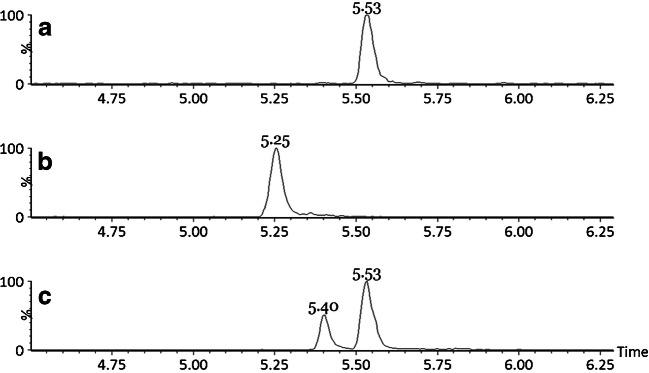
Chromatogram of plasma IGF-1 (**a**, 237.8 ng/mL), IGF-2 (**b**, 371 ng/mL), ^15^N-IGF-1 (**c**)

Representative chromatograms of IGF-1, IGF-2 and ^15^N-IGF-1 are presented in Fig. 2.

### Internal standard and calibration matrix

We used ^15^N-labelled human IGF-1 as internal standard in order to compensate for the variability arising from both the sample preparation and the LC-MS/MS analysis. As depicted in Fig. 2, ^15^N-IGF-1 gave rise to a second peak with a shorter retention time. Reduction and alkylation of the disulphide bonds resulted in a single peak for ^15^N-IGF-1 (see Supplementary Information (ESM) Fig. S6), indicating the presence of two proteoforms differing in their disulphide bond configuration. This finding is in line with similar observations in previous studies [22]. Given that no fully stable-isotope-labelled form of IGF-2 was available, ^15^N-IGF-1 was also used as internal standard for IGF-2. Although the peak in the ^15^N-IGF-1 chromatogram eluting at 5.40 min resembled IGF-2 more closely in terms of retention time, the peak at the retention time of IGF-1 was selected as the internal standard peak for IGF-2, as this peak corresponded with the disulphide bond configuration of endogenous IGF-1.

As IGF-free human plasma is not commercially available, we studied the effects of various surrogate matrices for calibration purposes. A high degree of similarity between patient matrix and calibration matrix was required in order to minimize variability during the precipitation steps. In the end, rat plasma was selected as calibration matrix, as its components, notably rat IGF-1 and IGF-2, did not interfere with endogenous human IGF measurement and no differences in retention times were observed.

### Assay characteristics

**Fig. 3:**
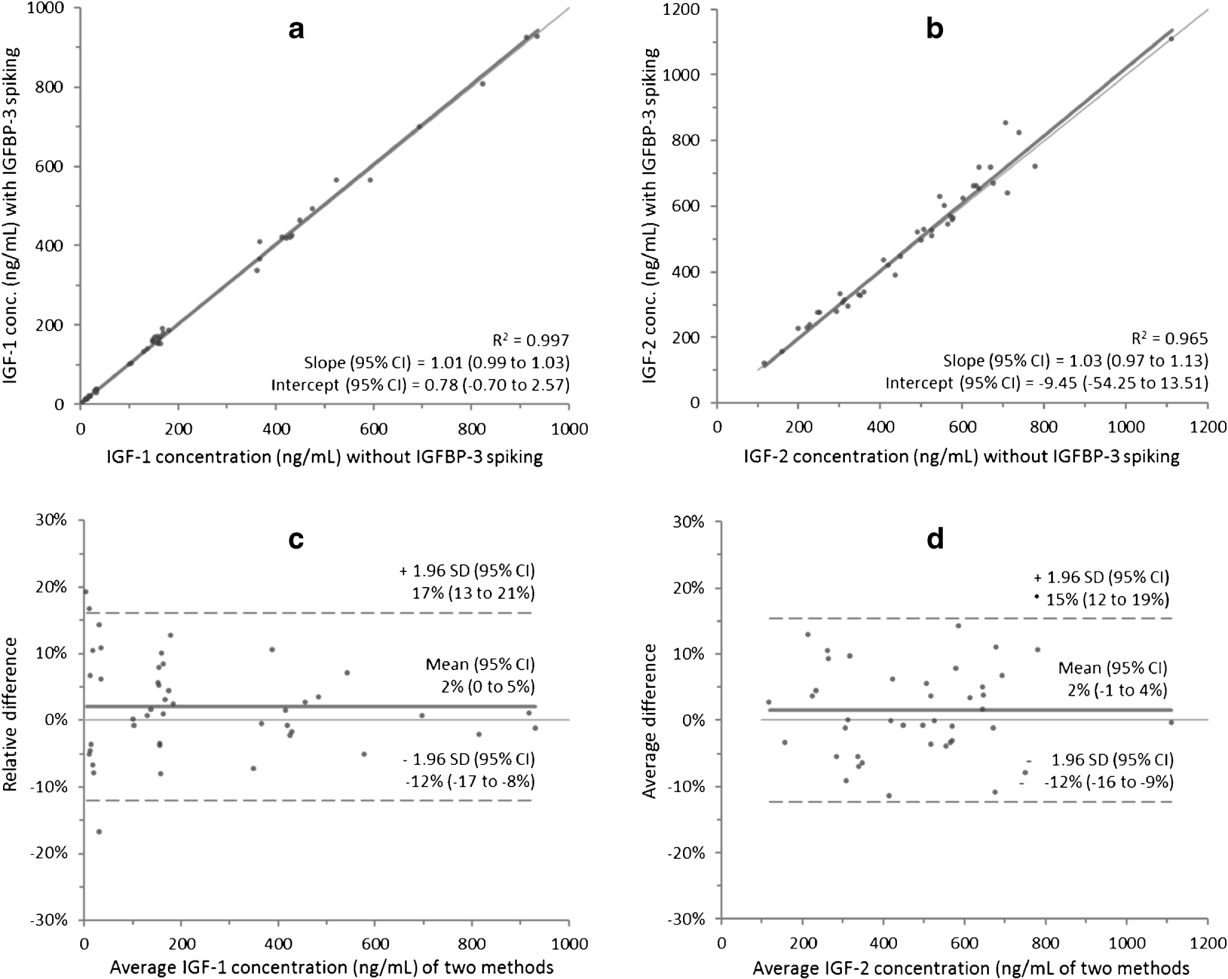
IGFBP-3 challenge test for IGF-1 (**a**, **c**) and IGF-2 (**b**, **d**) using Passing-Bablok regression and Bland-Altman plots

In the method detailed here, IGF/IGFBP complex dissociation is carried out by the addition of 50% TFE during sample preparation. In order to assess the effectiveness of this step, an IGFBP-3 challenge test was performed by spiking 45 patient samples with an excess of IGFBP-3. IGFBP-3 spiking did not result in the introduction of a significant bias (2.1% for IGF-1 and 1.5% for IGF-2, Fig. 3) into the method, thereby demonstrating the effectiveness of the use of TFE for IGF/IGFBP complex dissociation.

BCA analysis following the complete sample preparation workflow showed a total protein concentration of 5.0 mg/mL in 90 μL 1% FA in 10% acetonitrile, resulting in a total of approximately 25 μg of protein on column per injection. Despite this, column life well exceeded 1000 injections.

### Method validation and comparison

No significant ionisation suppression effects were present around the retention times where IGF-1 and IGF-2 eluted from the chromatographic column (ESM Fig. S7). Calibration curves of both IGF-1 and IGF-2 were linear across the calibration ranges with correlation coefficients (*R*^2^) > 0.99. No matrix or carryover effects were observed. Lower limits of quantification were determined at 5.9 ng/mL and 8.4 ng/mL for IGF-1 and IGF-2, respectively. Intra-assay coefficients of variation (CVs) were below 5% for IGF-1 and below 4% for IGF-2 at three QC levels, and inter-assay CVs were below 6% for both IGF-1 and IGF-2 at the three QC levels. Mean recoveries at three levels ranged from 94 to 97% for IGF-1 and 107–119% for IGF-2. Complete results for the intra- and inter-assay imprecision and the recovery of the method are presented in Table 2. Following sample preparation and when placed in the autosampler, both IGF-1 and IGF-2 were demonstrated to remain stable up to 7 days at 10 °C. A comprehensive overview of the validation results is included in ESM Tables S1–S5 and Figs. S8 and S9.

**Table 2:** Intra- and inter-assay imprecision and recoveries for IGF-1 and IGF-2

	Intra-assay			Inter-assay			Recovery		
	Mean (ng/mL)	SD (ng/mL)	CV (%)	Mean (ng/mL)	SD (ng/mL)	CV (%)	Mean (%)	SD (%)	CV (%)
IGF-1									
Low	55.2	1.0	1.8	56.6	2.3	4.0	97	11.1	11.5
Medium	255.6	7.8	3.0	251.6	10.1	4.0	94	5.2	5.5
High	638.5	28.7	4.5	631.5	34.4	5.5	95	7.4	7.7
IGF-2									
Low	458.7	10.9	2.4	450.3	26.0	5.8	108	5.8	5.4
Medium	1109.6	41.9	3.8	1089.5	56.8	5.2	109	9.8	9.0
High	1428.6	43.8	3.1	1379.1	78.6	5.7	107	8.9	8.3

WHO international standard 02/254 for IGF-1 was reconstituted in 2% (w/v) ovalbumin in PBS, diluted to 850 ng/mL and 85 ng/mL in rat plasma, and analysed according to the LC-MS/MS method detailed here. Mean measured concentrations were 853 ng/mL (bias 0.4%, CV 6.6%) and 85 ng/mL (bias − 0.5%, CV 3.9%), respectively.

**Fig. 4:**
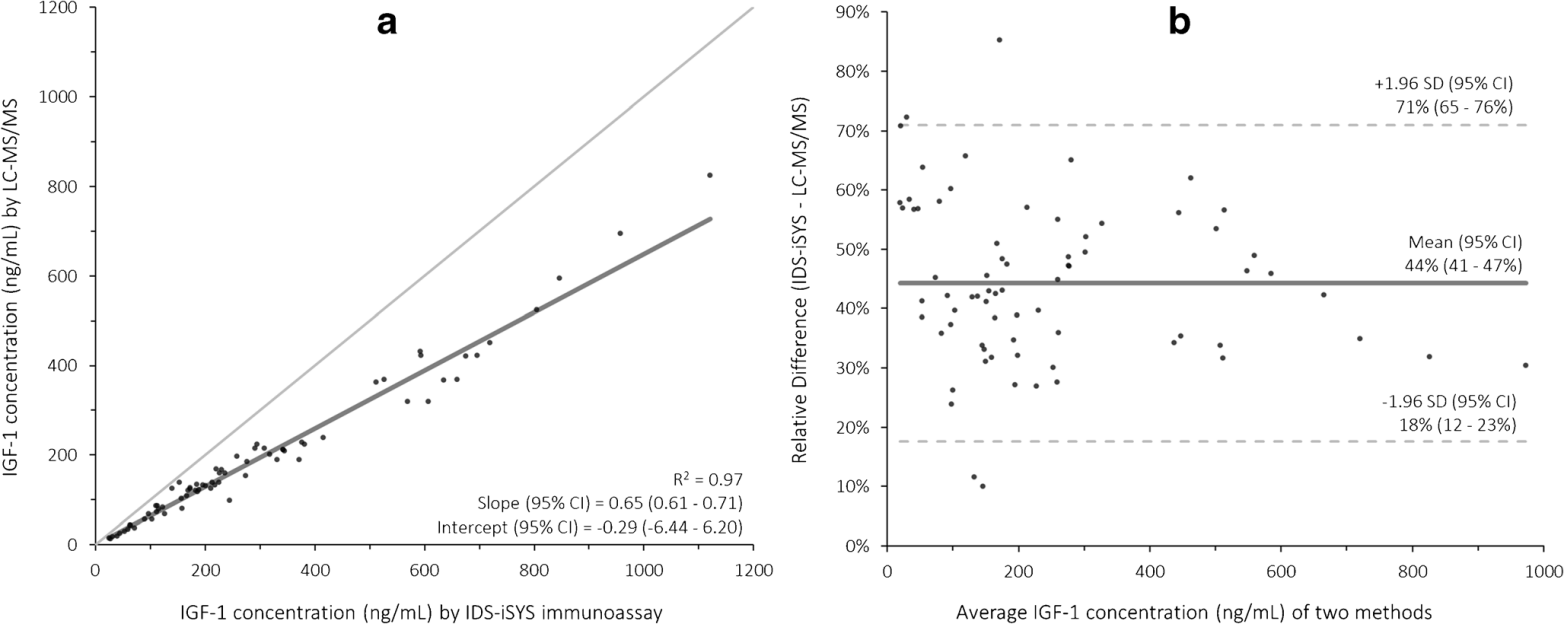
Comparison between the IGF-1 LC-MS/MS method and the IDS-iSYS IGF-1 immunoassay using Passing-Bablok (**a**) regression and Bland-Altman plots (**b**)

For IGF-1, a comparison was performed between the LC-MS/MS method detailed here and the IDS-iSYS immunoassay using 72 clinical samples (Fig. 4). Although the methods correlate well throughout the concentration range (*R*^2^ > 0.97), a significant bias was observed with the immunoassay yielding higher concentrations compared to the LC-MS/MS method.

## Discussion

We present an LC-MS/MS method for the simultaneous quantification of IGF-1 and IGF-2 in human plasma capable of accurately measuring levels across the clinically relevant concentration range. Given that the method does not rely on complex and time-consuming immunoaffinity techniques and reductive, alkylating and enzymatic digestion steps, it can be readily applied in clinical mass spectrometry laboratories as an alternative to immunoassays that suffer from poor reproducibility and interlaboratory comparability. Although different LC-MS/MS methods have been described for IGF-1, these methods mostly rely on the analysis of signature peptides following reduction, alkylation and trypsin digestion [17–23]. The main disadvantages of this methodology are that enzymatic digestion steps are relatively time-consuming and that it does not provide direct information regarding the intact protein. Alternatively, the analysis of intact IGF-1 using accurate mass LC-MS [26, 28] or immunoaffinity enrichment followed by MALDI-TOF mass spectrometry [27, 29] has recently been described. However, such instrumentation may be less readily accessible than triple quadrupole MS in clinical laboratories.

Given that no enzymatic digestion was used, our workflow required the use of a protein-based internal standard. We used ^15^N-labelled human IGF-1 as internal standard as this provides the most accurate compensation during the entire analytical process. The analysis of IGF-2 is limited by the absence of a fully stable-isotope-labelled form of the analyte. Although the validation criteria are still met, the assay characteristics for IGF-2 would benefit from the use of fully stable-isotope-labelled IGF-2 as internal standard.

The method detailed here relies on the addition of 50% TFE for IGF/IGFBP complex dissociation, the efficacy of which was demonstrated by the accuracy of IGF-1 and IGF-2 analysis in the presence of excess IGFBP-3. During method development, the addition of 8 M urea was also explored for dissociation. However, this resulted in increasing column back pressures within series and decreased within-run precision compared to TFE due to diminished signal intensity. Ultimately, TFE-based IGF/IGFBP complex dissociation presents an alternative to SDS, urea and acidification-based carrier protein dissociation methods.

For sample clean-up, a quick and simple two-step selective protein precipitation approach is introduced. By means of this workflow, we are able to remove approximately 90% of plasma proteins, while retaining smaller proteins in the sample [35]. Furthermore, as the supernatant is removed following the second precipitation step, the majority of potentially interfering salts and lipids are separated from the analytes. These factors all result in an interference-free chromatogram and no ionisation suppression around the retention times of IGF-1 and IGF-2, thereby allowing for sufficiently sensitive and robust quantification across the clinically relevant concentration ranges for IGF-1 and IGF-2 of 15–1100 ng/mL and 25–2000 ng/mL, respectively [36, 37].

The selective protein precipitation workflow is not only suitable for the analysis of IGFs, but can be easily adapted for clean-up for the analysis of similarly sized peptide hormones. As IGF-1 and IGF-2 are present in the blood at relatively high concentrations, though, other analytes might require additional sample preparation steps in order to reach sufficient analytical sensitivity. By careful selection and optimisation of the precipitation solvents, a selective protein precipitation approach might even have the potential to serve as an enrichment step at the protein level during the analysis of high-molecular mass proteins.

Analysis of the WHO international standard 02/254 for IGF-1 showed an excellent agreement between the measured and nominal concentrations. However, significantly lower concentrations of IGF-1 are found using the LC-MS/MS method described here compared to the IDS-iSYS IGF-1 immunoassay. This bias is in line with previous studies comparing intact IGF-1 methods with immunoassays and may be the result of different principles of analysis [27, 28]. Whereas IGF-1 concentrations obtained using the LC-MS/MS method detailed here are based solely on the detection of the intact protein at 7649 Da, immunoassays might measure a signal originating not only from the intact protein, but also from different proteoforms or degradation products.

The developed method is able to discriminate wild-type IGFs from different proteoforms and, therefore, the results obtained using this method relate to single defined IGF-1 and IGF-2 proteoforms, with specific physiological potencies. Given the characteristics of the sample treatment in combination with mass spectrometric detection, the method also allows for the monitoring of other known proteoforms, such as des(1,3)IGF-1 and oxidation products. As immunoassays, and to a lesser degree LC-MS/MS methods based on enzymatic digestion, might not be able to distinguish wild-type IGFs from proteoforms, they cannot guarantee this level of specificity. At the same time, this specificity might also be a limitation of the assay, as these structurally similar proteoforms might show biological activity.

In summary, we present a fully validated LC-MS/MS method for the quantification of intact IGF-1 and IGF-2 in human plasma that can be readily implemented in clinical mass spectrometry laboratories. The method introduces trifluoroethanol-based dissociation of IGF/IGFBP complexes and selective precipitation for sample clean-up, both of which may be applicable to the analysis of different peptide hormones.

## Supplementary Information

ESM 1 (PDF 1048 kb)

## Data Availability

Not applicable.
