# Supplementary material for: An antibody-free LC-MS/MS method for the quantification of intact insulin-like growth factors 1 and 2 in human plasma
Source: Anal Bioanal Chem. 2021 Feb 10;413(8):2035–44. doi: 10.1007/s00216-021-03185-y (PMC7943504; doi:10.1007/s00216-021-03185-y)
Supplement: Supplementary file 1 — (PDF 1048 kb) [file 216_2021_3185_MOESM1_ESM.pdf]

Supplementary Information

Analytical and Bioanalytical Chemistry

An antibody-free LC-MS/MS method for the quantification of intact insulin-like growth factors 1 and 2 in human plasma

Mark S. Pratt, Martijn van Faassen, Noah Remmelts, Rainer Bischoff and Ido P. Kema

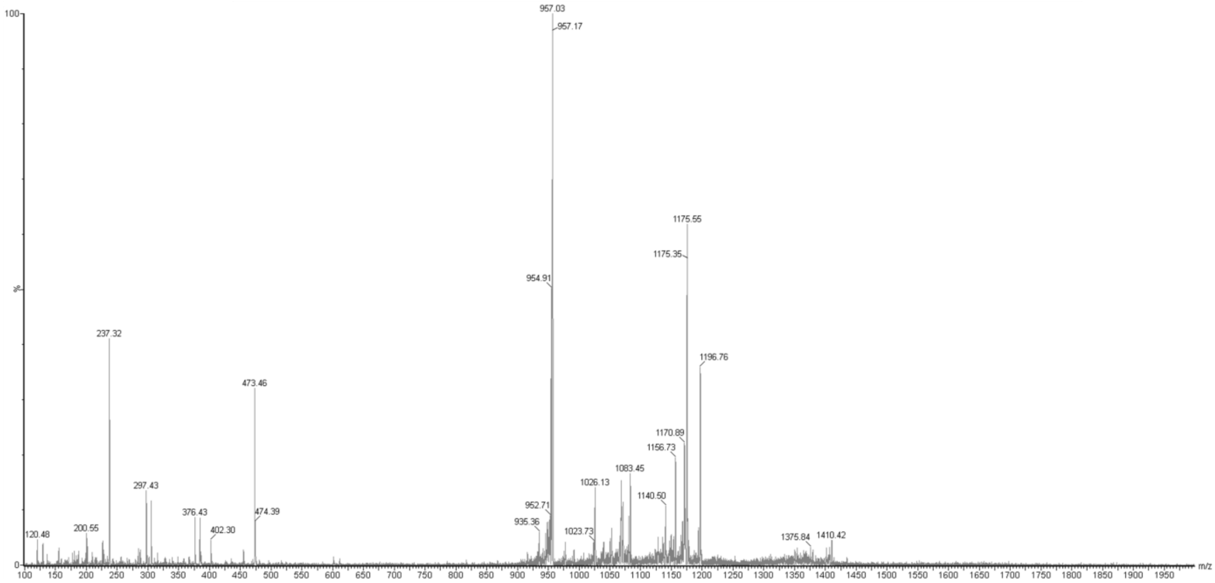

Fig. S1: Product ion scan for precursor ion m/z 956.95.
Amino acid sequence precursor ion: GPETLCGAEL VDALQFVCGD RGFYFNKPTG YGSSSRRAPQ TGIVDECCFR SCDLRRLEMY CAPLKPAKSA (IGF-1 – $[M+8H]^{8+}$)
Amino acid sequence product ion m/z 1175.55: GPETLCGAEL VDALQFVCGD RGFYFNKPTG YGSSSRRAPQ TGIVDECCFR SCDLRRLEMY CAPL (b_{64}^{6+})

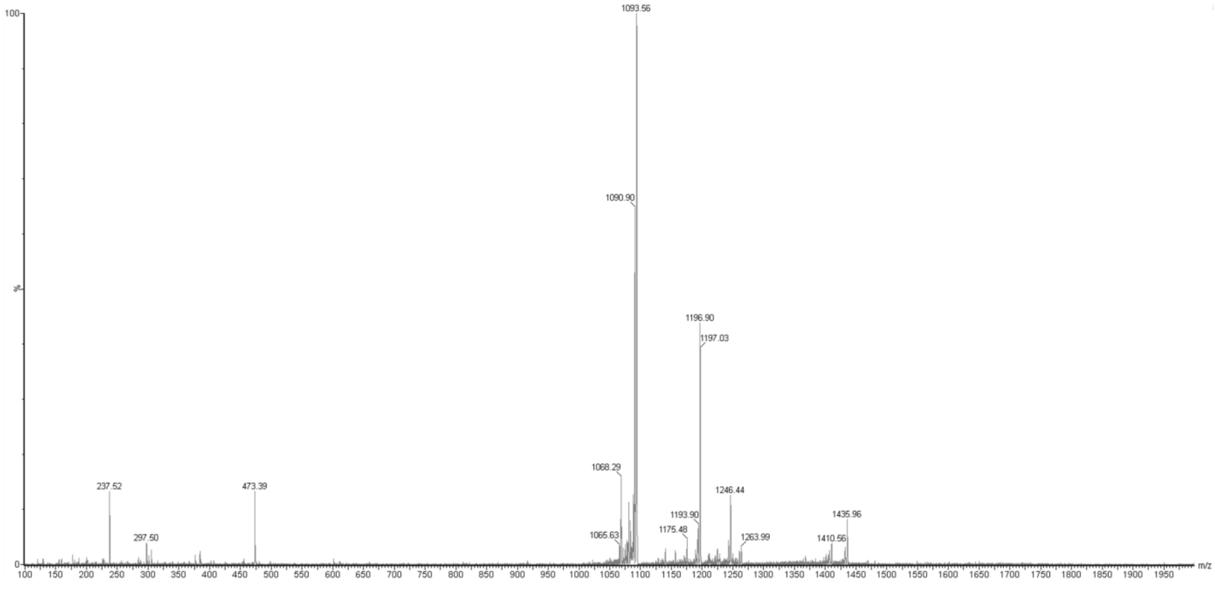

Fig. S2: Product ion scan for precursor ion m/z 1093.45.
Amino acid sequence precursor ion: GPETLCGAEL VDALQFVCGD RGFYFNKPTG YGSSSRRAPQ TGIVDECCFR SCDLRRLEMY CAPLKPAKSA (IGF-1 – $[M+7H]^{7+}$)
Amino acid sequence product ion m/z 1196.95: GPETLCGAEL VDALQFVCGD RGFYFNKPTG YGSSSRRAPQ TGIVDECCFR SCDLRRLEMY CAPLK (b_{65}^{6+})

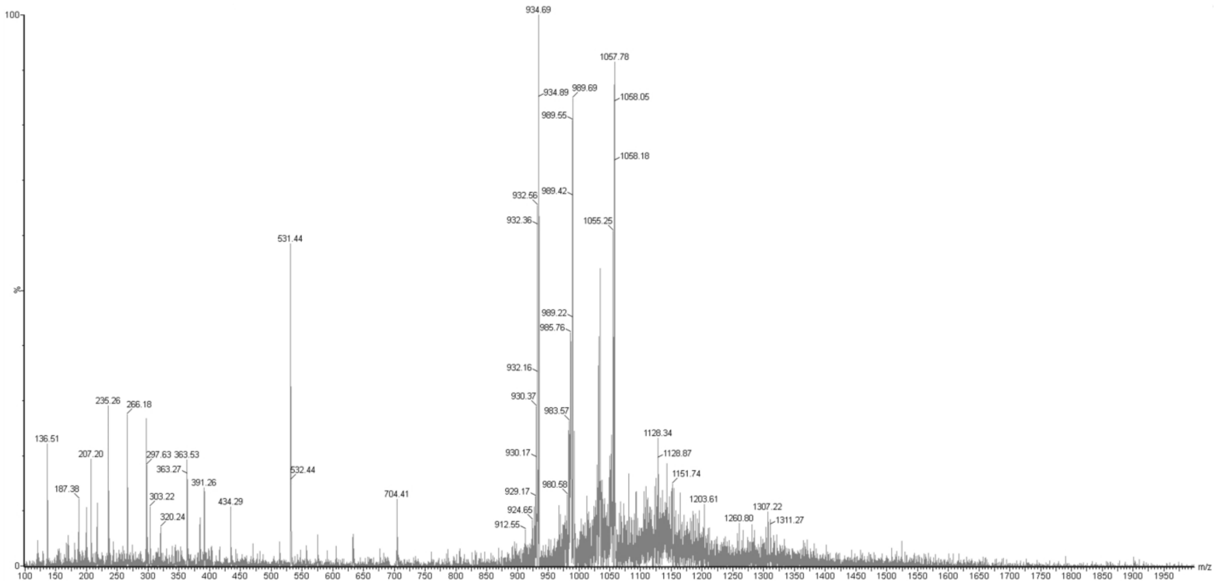

Fig. S3: Product ion scan for precursor ion m/z 934.50.
Amino acid sequence precursor ion: AYRPSETLCG GELVDTLQFV CGDRGFYFSR PASRVSRRSR GIVEECCFRS CDLALLETYC ATPAKSE (IGF-2 – $[M+8H]^{8+}$)
Amino acid sequence product ion m/z 1057.90: YRPSETLCGG ELVDTLQFVC GDRGFYFSRP ASRVSRRSRG IVEECCFRSC DLALLETYCA TPAKSE (y_{66}^{7+})
Amino acid sequence product ion m/z 989.70 ETLCGGELVD TLQFVCGDRG FYFSRPASRV SRRSRGIVEE CCFRSCDLAL LETYCATPAK SE (y_{62}^{7+} - H_2O)

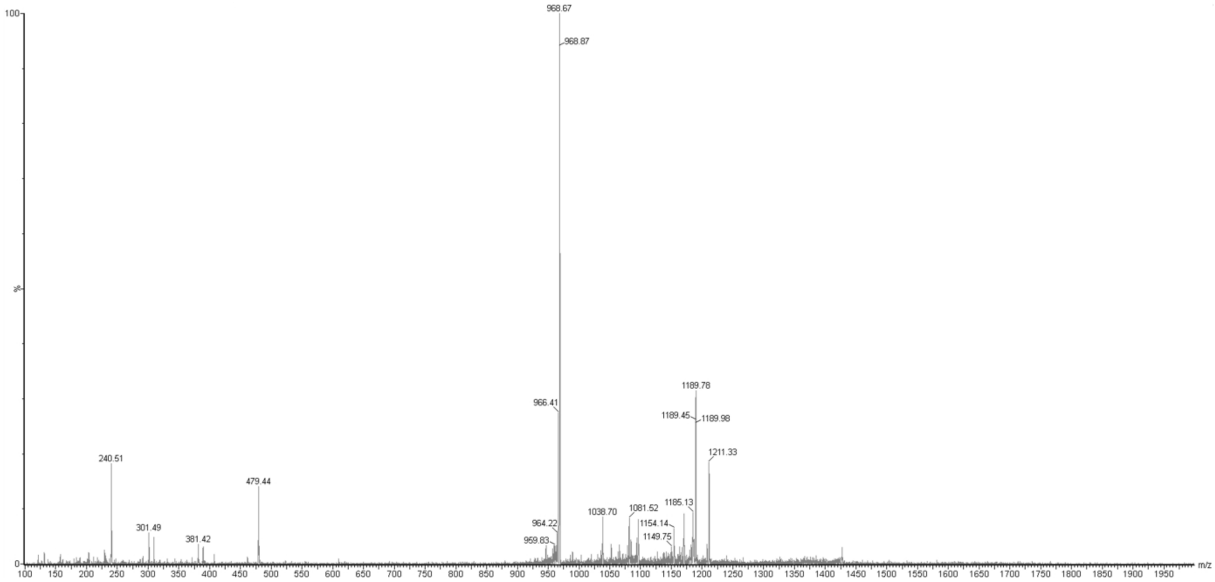

Fig. S4: Product ion scan for precursor ion m/z 968.50.
Amino acid sequence precursor ion: GPETLCGAEL VDALQFVCGD RGFYFNKPTG YGSSSRRAPQ TGIVDECCFR SCDLRRLEMY CAPLKPAKSA (15N-IGF-1 – $[M+8H]^{8+}$)
Amino acid sequence product ion m/z 1189.70: GPETLCGAEL VDALQFVCGD RGFYFNKPTG YGSSSRRAPQ TGIVDECCFR SCDLRRLEMY CAPL (b_{64}^{6+})

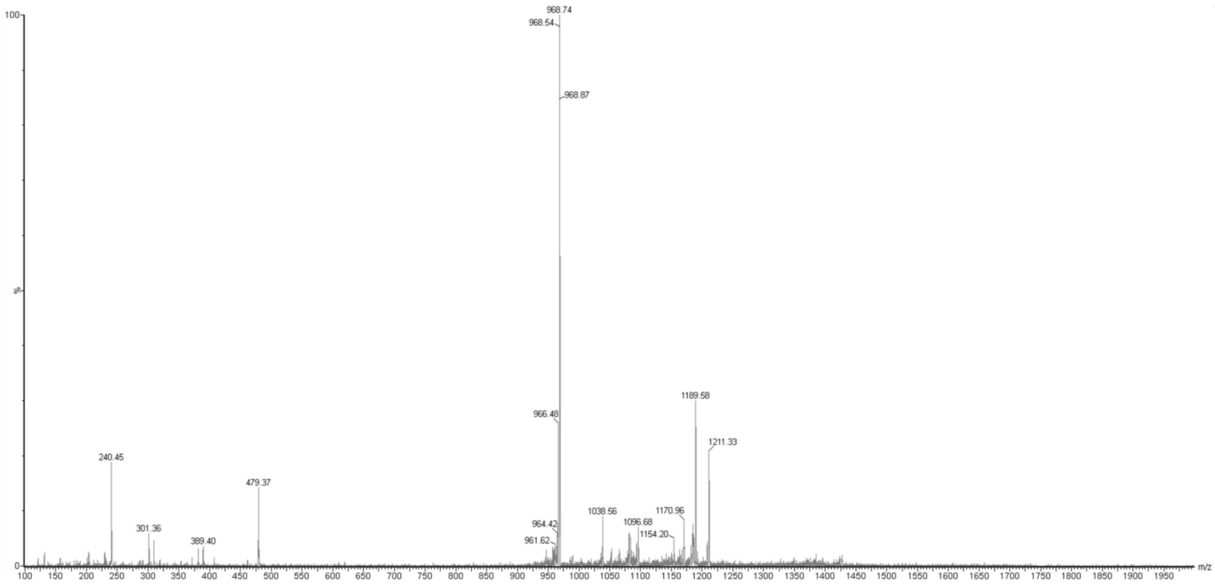

Fig. S5: Product ion scan for precursor ion m/z 1106.65.
Amino acid sequence precursor ion: GPETLCGAEL VDALQFVCGD RGFYFNKPTG YGSSSRRAPQ TGIVDECCFR SCDLRRLEMY CAPLKPAKSA (15N-IGF-1 – $[M+7H]^{7+}$)
Amino acid sequence product ion m/z 1211.45: GPETLCGAEL VDALQFVCGD RGFYFNKPTG YGSSSRRAPQ TGIVDECCFR SCDLRRLEMY CAPLK (b_{65}^{6+})

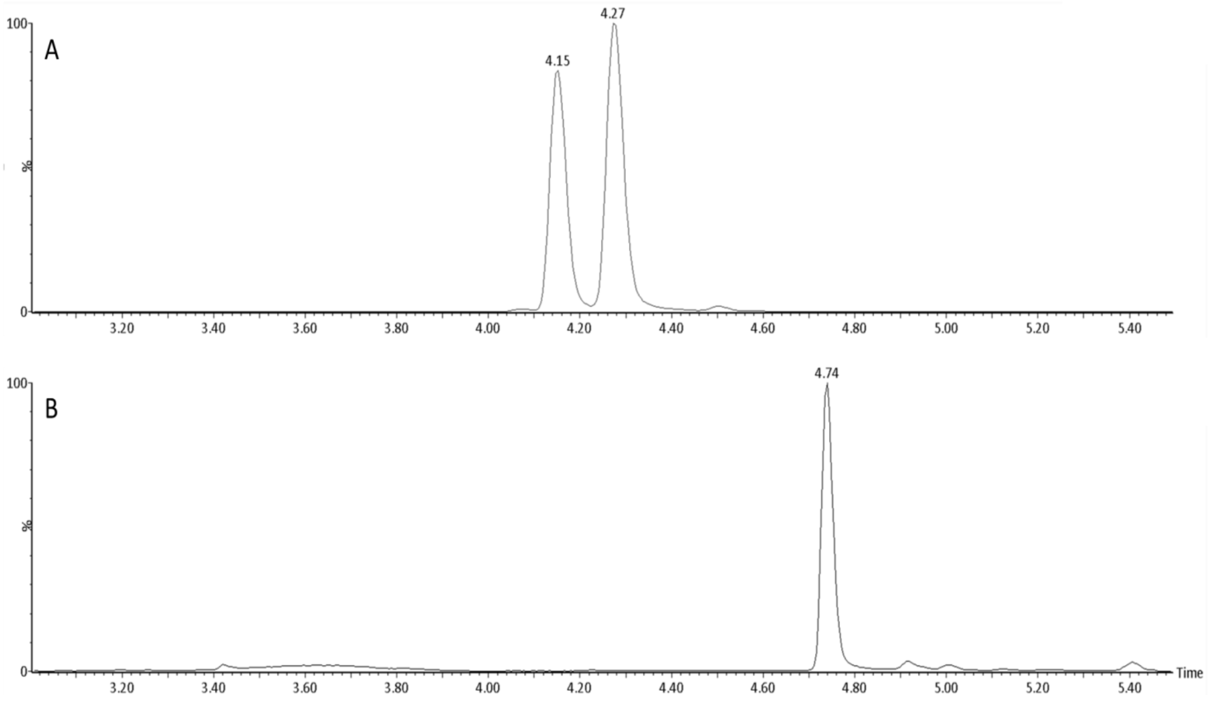

Fig. S6: Chromatogram of ^{15}N-IGF-1 (A) and ^{15}N-IGF-1 following reduction by means of dithiothreitol and alkylation by means of iodoacetamide (B).

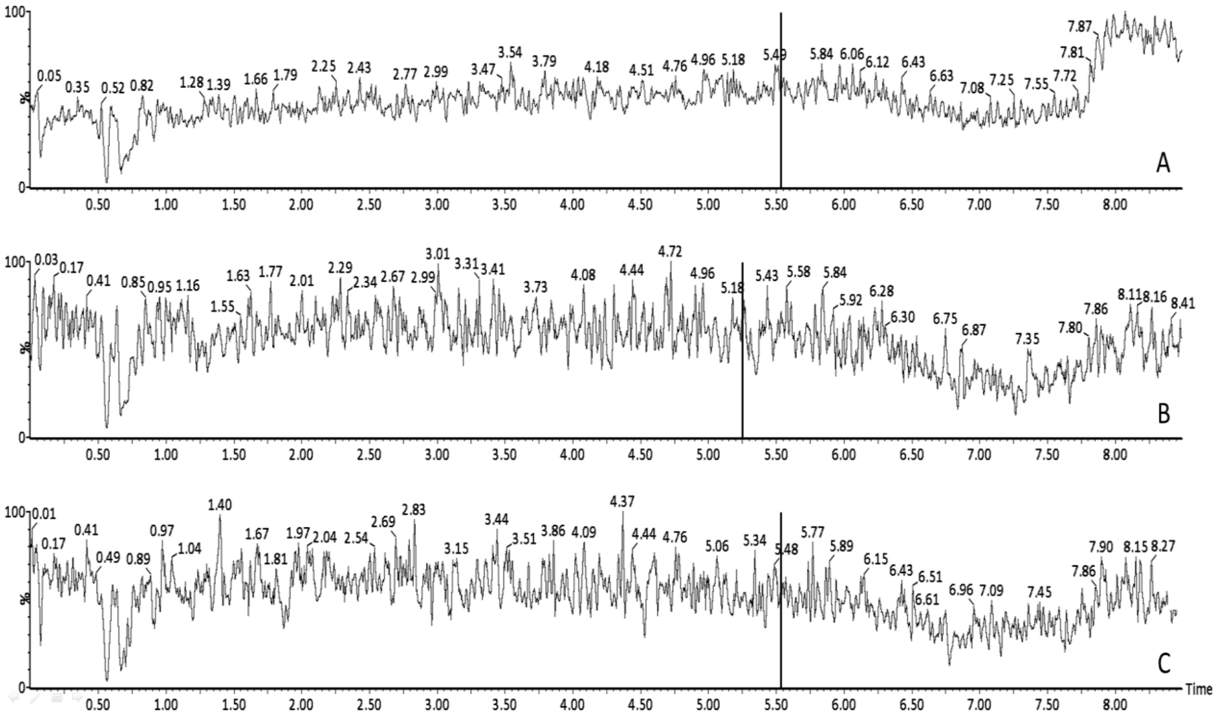

Fig. S7: Post-column infusion chromatograms for IGF-1 (A), IGF-2 (B) and ^{15}N-IGF-1 (C) after injection of a plasma sample with low IGF-1 and IGF-2 concentrations. The bars in the chromatograms represent the retention times of the respective analytes.

Table S1: Matrix effect validation results for IGF-1.

	High sample (%)	Mean conc. (ng/mL)
100% low sample	0	58.3
75% low sample, 25% high sample	25	210.4
50% low sample, 50% high sample	50	344.2
25% low sample, 75% high sample	75	498.3
100% high sample	100	665.2

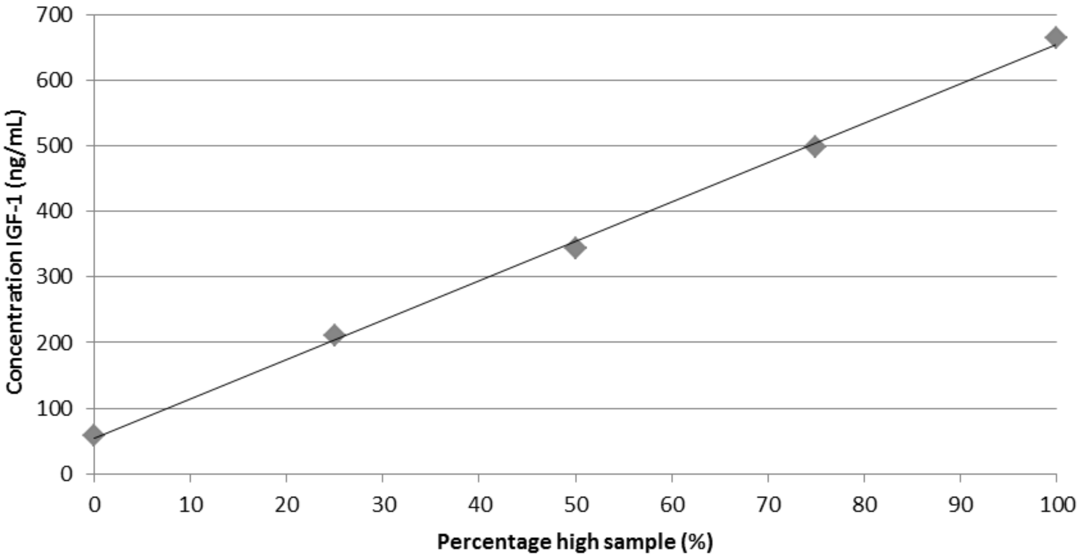

Fig. S8: Matrix effect curve for IGF-1.
R^2 = 0.999.

Table S2: Matrix effect validation results for IGF-2.

	High sample (%)	Mean conc. (ng/mL)
100% low sample	0	465
75% low sample, 25% high sample	25	705
50% low sample, 50% high sample	50	920
25% low sample, 75% high sample	75	1212
100% high sample	100	1424

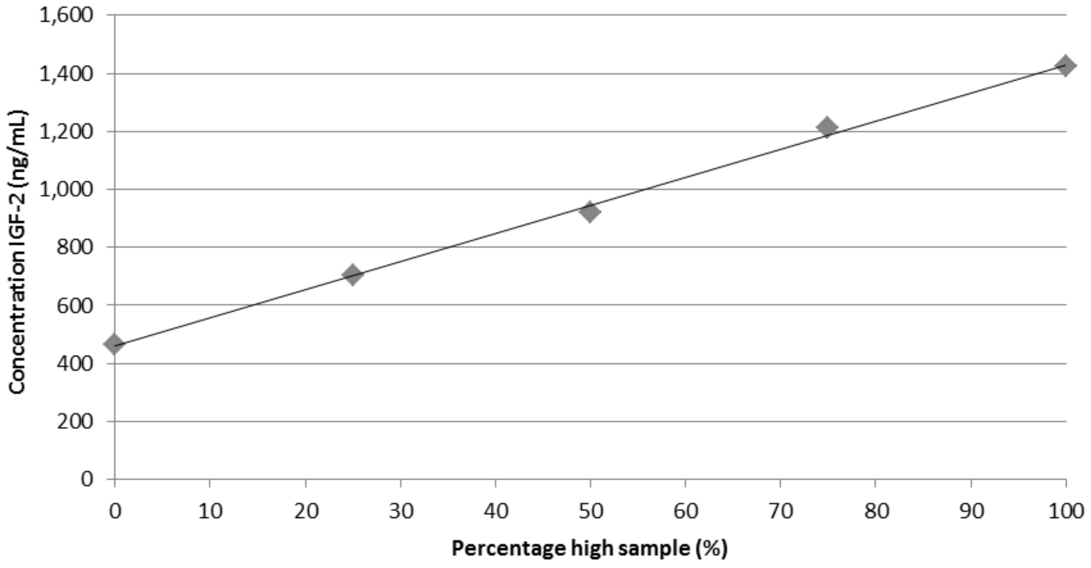

Fig. S9: Matrix effect curve for IGF-2.
R^2 = 0.998.

Table S3: Linearity validation results for IGF-1 and IGF-2 across six days.

	L2	L3	L4	L5	L6	L7	L8	Slope	R^2
**IGF-1**									
Mean (ng/mL)	14.3	29.1	60.3	182.5	365.2	742.6	1160.4	0.00691	0.9979
SD (ng/mL)	0.8	0.8	2.6	5.0	6.7	13.0	19.6		
CV (%)	5.4	2.8	4.3	2.7	1.8	1.8	1.7		
**IGF-2**									
Mean (ng/mL)	19	40	85	276	495	958	1703	0.00254	0.9943
SD (ng/mL)	1.7	2.1	4.0	8.4	17.8	18.7	34.0		
CV (%)	9.0	5.3	4.7	3.1	3.6	2.0	2.0		

Table S4: Autosampler stability validation data for IGF-1 and IGF-2 up to 7 days.

	Mean (ng/mL)	SD (ng/mL)	CV (%)		Mean (ng/mL)	SD (ng/mL)	CV (%)
**IGF-1**				**IGF-2**			
Sample 1	114.7	6.3	5.5	Sample 1	466	28.9	6.2
Sample 2	223.7	9.3	4.0	Sample 2	737	21.9	3.0
Sample 3	105.7	4.2	4.0	Sample 3	604	28.2	4.7
Sample 4	118.8	4.5	3.8	Sample 4	623	21.8	3.5
Sample 5	141.2	6.4	4.5	Sample 5	536	18.8	3.5
Sample 6	68.6	3.1	4.6	Sample 6	490	20.5	4.2

Table S5: Individual results from tenfold analysis of the NIBSC reference standard 02/254 for IGF-1.

Nominal Concentration	Measured concentration (ng/mL)					Mean (ng/mL)	Bias (%)	CV (%)
85 ng/mL	85.4	84.6	81.3	82.2	89.6	84.6	-0.5	3.9
	78.2	84.2	83.8	85.4	90.4			
850 ng/mL	732.7	871.5	884.6	917.9	874.7	853.4	0.4	6.6
	790.8	878.4	867.0	913.7	890.2			
